# Behavioral assessment and gene expression changes in a mouse model with dysfunctional STAT1 signaling

**DOI:** 10.1186/s12964-025-02313-w

**Published:** 2025-07-01

**Authors:** Luca Büschgens, Nina Hempel, Aditi Methi, Andre Fischer, Nele Siering, Lars Piepkorn, Olaf Jahn, Thomas Meyer, Oliver Wirths

**Affiliations:** 1 Department of Psychiatry and Psychotherapy, University Medical Center (UMG), Georg-August-University, Von-Siebold-Str. 5, 37075 Göttingen, Germany; 2 German Center for Neurodegenerative Diseases (DZNE), Göttingen, Germany; 3 Neuroproteomics Group, Department of Molecular Neurobiology, Max Planck Institute for Multidisciplinary Sciences, Göttingen, Germany; 4 Department of Psychosomatic Medicine and Psychotherapy, University Medical Center (UMG), Georg-August-University, Göttingen, Germany

**Keywords:** STAT signaling, Interferon signaling, Gene expression, Proteome analysis, Behavior, Mice

## Abstract

**Background:**

Signal transduction via the Signal Transducer and Activator of Transcription 1 (STAT1) pathway is indispensable for mediating the intracellular effects of interferon-α (IFN-α), interferon-γ (IFN-γ) and other cytokines in the brain and, thereby, crucial for antiviral and antibacterial responses during potential life-threatening CNS infections. However, the role of STAT1 signaling beyond the known IFN-α and IFN-γ effects in immediate antimicrobial defense is highly context-dependent, and studies in the existing literature using STAT1-targeted mouse models under normal physiological conditions remain scarce.

**Methods:**

Here, we characterized a STAT1 targeted-disruption mouse model in the absence of infectious stimuli by employing established behavioral testing paradigms and immunohistochemical stainings, as well as bulk hippocampal transcriptomic and proteomic analyses.

**Results:**

While we found neither overt behavioral alterations nor immunohistochemical changes with respect to microglial phagocytosis or proliferation, significant alterations were detected in gene and protein expression profiles implicated in neuroinflammatory processes and neuroprotection.

**Conclusion:**

In summary, this study highlights the complex and context-dependent role of STAT1-mediated signaling even in the absence of any detectable behavioral and neuropathological changes.

**Supplementary Information:**

The online version contains supplementary material available at 10.1186/s12964-025-02313-w.

## Introduction

**Fig. 1:**
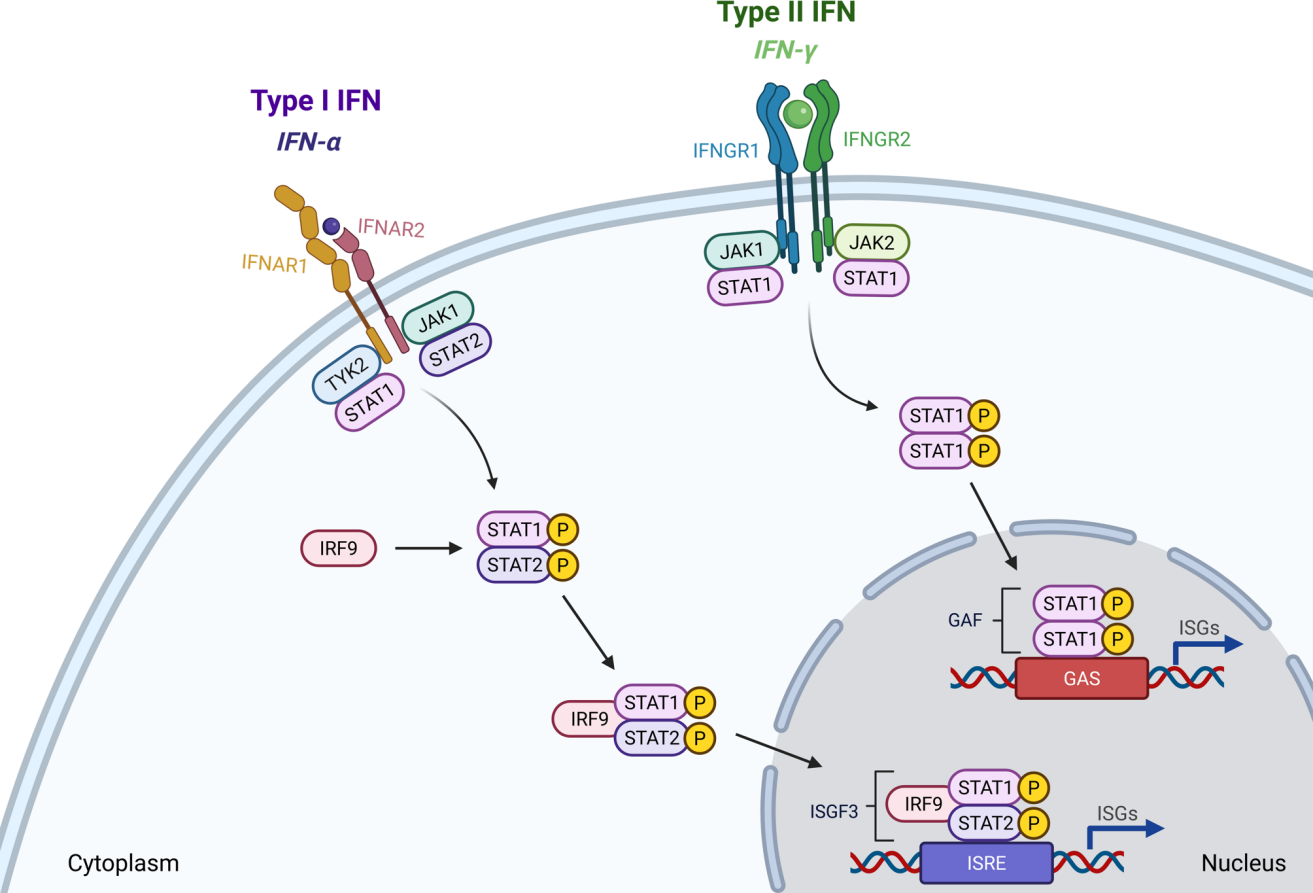
Overview of the Jak-STAT signaling pathway. Upon binding of cytokines and growth factors, receptor dimerization leads to JAK recruitment, subsequent tyrosine phosphorylation and STAT docking site formation. Following tyrosine phosphorylation, STATs separate from the receptor and form homo- or heterodimers prior to entering the nucleus and activating transcription of target genes. IFNAR – Interferon-α receptor; IFNGR – Interferon-γ receptor; JAK – Janus kinase; GAS – Gamma interferon activation site; ISRE – interferon-α stimulated response element; ISG – Interferon-stimulated gene. Created in BioRender. Büschgens, L. (2025) https://BioRender.com/ylfsaz1

The Janus Kinase/Signal Transducer and Activator of Transcription (JAK/STAT) pathway, a signaling mechanism that is evolutionarily highly conserved, plays a crucial role in multiple physiological processes such as cell survival, inflammation, oncogenesis, and hematopoiesis [1, 2]. It allows cells to respond to extracellular signals and serves as a direct route from the cytoplasmic membrane to transcriptional regulation in the nucleus. The JAK/STAT pathway consists of a combination of core components located at three hierarchical levels, namely structurally diverse cytokine receptors, four different Janus kinases (JAK1, JAK2, JAK3 and TYK2) and in humans seven members of the STAT family (STAT1, STAT2, STAT3, STAT4, STAT5A, STAT5B and STAT6) [3]. Upon binding of extracellular proteins such as growth factors, cytokines and interferons to the ligand-binding subunit, the respective cytokine receptors undergo dimerization or oligomerization and, thereby, bring the receptor-associated Janus kinases into close proximity [4] (Fig. 1). This clustered recruitment of JAKs then facilitates their transphosphorylation enabling their kinase activity to phosphorylate in turn carboxy-terminal tyrosine residues in the intracellular receptor chains. The phosphorylated receptors subsequently create docking sites for the STAT proteins. Once the STATs are bound, they are phosphorylated by the JAKs, which initiate the formation of tyrosine-phosphorylated, parallel-oriented STAT dimers. The phosphorylated STAT dimers are then able to enter the nucleus to facilitate the transcription of a plethora of target genes [5]. Although STAT1 is known for its tumor suppressive functions [2], it plays an even greater role in immune antiviral and inflammatory responses upon interferon-α (IFN-α, type I interferon) and IFN-γ (type II interferon) activation [6]. Depending on its activating cytokine, STAT1 forms hetero- or homo-dimers to initiate different transcriptional programs inside the nucleus [7]. In the central nervous system (CNS), IFN-α and IFN-γ signaling differentially influence inflammatory environment and cognitive function, thereby leading to either detrimental or beneficial effects depending on the context and cytokine concentration [8, 9]. Thus, the existence of many conflicting conclusions on the effects of cytokines in the brain is not surprising.

One mode of action of IFN-γ in affecting cognition has been reported to be its ability to influence hippocampal neurogenesis. In fact, several studies have shown that chronic IFN-γ administration is linked to reduced hippocampal neurogenesis [10], mainly by acting as an inhibitor of proliferation [11] of neural stem/precursor cells (NSPCs) and by inducing reactive phenotypes in microglia that are associated with pro-inflammatory cytokine and reactive-oxygen species (ROS) production [12]. Conversely, inhibiting IFN-γ production or modulating its effects on microglia specifically resulted in improvements in hippocampus-dependent cognitive functions [10]. However, the neurogenesis-inhibiting effect of IFN-γ also seems to depend on physiological conditions, such as the presence or absence of a stressor or a clearly defined pathological trigger. While baseline IFN-γ production and signaling appear to support memory function as shown in reports providing evidence for a neuroprotective effect under normal conditions [13], an up-regulated, chronic IFN-γ production under neuroinflammatory and pathological conditions, as found for example in neurodegenerative diseases, seems to result in deleterious effects [9].

Likewise, several studies investigating the effect of augmented and prolonged IFN-α production reported a decreased hippocampal neurogenesis and increased inflammatory cytokine production [14] as well as concomitant neurodegeneration [15]. IFN-α plays a pivotal role in immediate antiviral response [16] and is widely used as treatment for viral infectious diseases such as hepatitis C [17]. On the other hand, constantly elevated levels are associated with adverse effects such as dementia as seen, for example; in HIV-associated neurocognitive disorders [18, 19], neuropsychiatric side effects such as depression [20], as well as in transgenic models of progressive inflammatory encephalopathy [15], and synaptic dysfunctions in a transgenic Alzheimer’s disease (AD) mouse model [21].

Both IFN-α and IFN-γ signaling affect multiple cell types and cell-cell-interactions in the CNS, with microglia being one of the major effector cells particularly involved in neuroinflammatory responses and related diseases [21, 22]. The processes initiated by both IFN types can acquire an enhancing or alleviating character, depending on the specific function, context, environment and additional stimuli, the microglia are exposed to [23]. This is further complicated by the various dynamic states that microglia can adopt, which are highly different depending on the pathologic situation [24]. Such processes include the increased production of ROS and pro-inflammatory cytokines (such as IL-6, TNF-α, IL-1α, IL-1β, and IL-12), production of inflammasome complexes, and T-cell recruitment as a first response to neutralize invading pathogens when stimulated with IFN-γ [25, 26]. In addition, synaptic engulfment and pruning, complement system activation, morphological changes termed hyper-ramification, and regulating T cell cytotoxicity have been described upon IFN-α stimulation [27, 28].

As mentioned above, numerous studies have reported on the beneficial properties of both IFN-α and -γ during viral infections as well as on detrimental effects by excessive administration or prolonged cytokine exposure during neuroinflammatory events or diseases. Hence, in the present study, we have investigated how elimination of both IFN-α and IFN-γ signaling through amino-terminal truncation of the STAT1 protein affects animal behavior, brain-specific transcriptome and proteome alterations as well as the microglial state under non-pathological conditions.

## Materials and methods

### Mouse model

In this study, a homozygous STAT1 targeted-disruption mouse model showing a complete lack of responsiveness to both IFN-α and IFN-γ [29] was used (Taconic Biosciences, #2045). In brief, this was achieved by inserting a STAT1-targeting construct to replace the first three translated exons of the STAT1 gene. This line, which was used on a mixed C57Bl6/N and C57BL6/J genetic background in the current study and kept as a homozygous line, produces only a low level of abnormal and functionally inactive STAT1 protein (in the following designated as STAT1^−/−^). Apart from the lack of IFN signaling, response to other cytokines is retained and the mice do not show any developmental abnormalities apart from a slightly increased susceptibility to opportunistic infections [29]. For behavioral testing, a matching number of female and male animals and age-matched WT mice (C57Bl6/J) of 3 and 7 months of age were used. Animals had unrestricted access to food and water throughout the study. Mice were kept in individually ventilated cages and all material was autoclaved prior use. All animals were handled in compliance with the German guidelines for animal welfare, with all experimental procedures approved by the local Animal Care and Use Committee (LAVES, Lower Saxony, Germany). All experiments followed the recommendations of the ARRIVE guidelines.

### Behavioral tests

In general, only one test per behavioral domain was conducted per day. On days involving both cognitive and motor assessments, testing always began with the cognitive task, followed by the motor task. Both male and female mice were used in all behavioral experiments, with efforts to ensure approximately equal representation of both sexes. To assess motor learning and coordination, the rotarod and the balance beam task were used. The rotarod experiment was conducted under standardized conditions using red light (~ 130 lx). All mice received a pre-test until falling from the rotating cylinder to familiarize with the rotating rod (TSE Systems, Bad Homburg, Germany). The test was conducted over two consecutive days with 4 trials per day for each mouse (intertest interval of 10 min), with a rod acceleration of 4 to 40 rpm over a maximum trial duration of 5 min. Each trial ended when the mice left the rod or managed to stay for the maximum duration of 5 min. The setup of the balance beam task consisted of a 1 cm wide beam with a length of 50 cm that was placed 44 cm above a padded surface. On each side of the beam, a 9 × 15 cm platform was attached. In a pre-test, the mice were familiarized with the beam by placing them in the middle and letting them reach one of the escape platforms. Each mouse received 3 trials with a maximum duration of 60 s and a minimum resting time of 10 min between each trial. Mice were placed in the middle of the beam and the time on the beam was measured. If mice managed to reach one of the platforms, the maximum time of 60 s was recorded and the average time of all three trials was taken as final score for each mouse.

The novel object recognition memory task was used to evaluate recognition memory, which relies on the natural attraction of rodents to novelty [30]. The experiment was conducted on three consecutive days and divided into Day 1: Habituation, Day 2: Training Day and Day 3: Testing Day. Habituation consisted of 5 min of free exploration in a standard open field test (50 × 50 cm arena). On the training day, two identical objects were presented in the arena for free exploration. On the third day, one of these objects was replaced with a novel one. A 20 mm investigation zone was defined around each object. Exploration was recorded whenever the mouse entered this zone with its head oriented toward the object. On both testing days, exploration time of both objects and the distances traveled were recorded via the ANY-Maze (Stoelting, Wood Dale, IL, USA) program for every mouse during 10 min trials. Recognition performance was assessed by the Discrimination Index (DI), calculated as the difference between the exploration times of the novel (T_novel_) and familiar (T_familiar_) objects, relative to the total exploration time (T_total_).

The Morris water maze (MWM) was used to evaluate spatial reference memory [31], following established protocols [32]. Briefly, mice first underwent three days of cued training, with each day consisting of four 60-sec trials separated by 10 min intervals. During cued training, the platform was marked with a triangular flag, and both the platform location and starting point varied across the four quadrants, followed by a five-day acquisition training, with four trials per day. In this phase, the flag was removed, and the platform remained in a fixed position for each mouse in all trials. Trials were separated by a minimum resting time of 10 min. Visual cues were available around the pool to aid spatial learning. Throughout both training phases, the time to reach the platform as well as the speed and distance travelled were recorded using an automated video tracking system (ANY-Maze). A probe trial, assessing spatial memory, was conducted 24 h after the last acquisition day. In this trial, the platform was removed, and mice were introduced from a new starting point. Mice swam freely for 60 s, and the time spent in each quadrant was recorded. During the MWM paradigm, no additional behavioral testing was performed.

In order to evaluate exploratory behavior and intensity of anxiety, the elevated plus maze task was carried out, as previously described [33]. The setup consists of four arms (15 × 5 cm) extending from a square center (5 × 5 cm) that arranged in an angle of 90° and is elevated 75 cm above a padded surface. Two arms are closed with 15 cm high walls of clear plastic and two arms are open. The experiment is conducted under red light (~ 130 lx). The mouse is placed in the square center facing one of the open arms and performs a single 5 min trial. Between each trial, the setup is cleaned with disinfectant to reduce odor cues.

The cross maze task was used to further assess spatial working memory indicated by spontaneous alternation rates, as previously described [33]. In brief, the setup consists of a maze with four arms (30 × 8 × 15 cm), arranged orthogonally from a square center (8 × 8 cm). Mice were placed in one of the arms facing the wall and were allowed to freely explore the maze under red light conditions. Each mouse received a single 10-min trial and arm entries were recorded automatically via the video tracking system. Raw data were analyzed using a custom Excel macro.

### Tissue collection and preservation

Immediately after completing behavioral testing, the mice were sacrificed by CO_2_ asphyxiation and subsequent cervical dislocation. The brain was dissected and hemispheres separated. Left hemispheres were stored in 4% buffered formalin (Roti Histofix, Carl Roth, Germany) for 6 days, dehydrated in an ascending ethanol series and embedded in paraffin. From the right hemispheres, cortices and hippocampi were isolated and immediately frozen on dry ice to be stored at -80 °C until further use.

### RNA isolation, cDNA synthesis & quantitative real-time PCR

RNA was isolated from deep frozen hippocampal and cortical tissue of male mice. The tissues were weighed and homogenized with 15 strokes at 800 rpm in a glass-teflon homogenizer after addition of 1 ml TRIzol reagent (#15596018, ThermoFisher Scientific) per 100 mg of sample. The homogenates were incubated at RT for 5 min for dissociation of nucleoprotein complexes. Then, 0.2 ml of chloroform was added to each ml of TRIzol and samples were vigorously vortexed for 15 s and incubated for 10 min at RT. Following a centrifugation step at 12,000 x g for 15 min at 4 °C, the upper aqueous RNA-containing phase was transferred into a fresh tube containing ice-cold (-20 °C) isopropanol (0.5 ml per 1 ml TRIzol). After 20 min of incubation at -20 °C, samples were centrifuged and washed with 100 µl of ice-cold 75% ethanol for 3 consecutive times. Pellets were air-dried at RT for at least 30 min until they became transparent, subsequently dissolved in 30 µl molecular grade H_2_O and incubated on ice for 1 h before being stored at -80 °C.

For cDNA synthesis and genomic DNA digestion, the RevertAid reverse transcription (K1691) and DNase 1 (EN0521) kits were used according to the manufacturer’s manual (ThermoFisher Scientific). The amount of RNA used was 1000 ng, with a final concentration of 50 ng/µl in the cDNA synthesis mastermix. The resulting cDNA was diluted 1:10 in sterile ddH_2_O for further use in qRT-PCR.

For gene expression analysis, qPCR was carried out with SYBR green as intercalating fluorescent dye (Blue’s Green qPCR Kit, Biozym, Germany) on a CFX Connect Real-Time PCR Detection System (BioRad, Feldkirchen, Germany). Samples were pipetted in duplicates and average CT values were calculated from raw data. Relative gene expression analysis was conducted, using murine *β-actin* as reference gene, and normalization to the control group was carried out using the Pfaffl method including efficiency correction [34]. Sequence and amplicon size information of primers used in this study can be found in Additional file 1.

### Fluorescent immunostaining

Sagittal brain sections of female mice (4 μm thickness) were prepared on a rotation microtome (Slee Medical, Mainz, Germany) and used for staining (starting from Bregma lateral 0.84). In brief, sections were deparaffinized using Roticlear (Carl Roth, Germany), and rehydrated in a descending ethanol series. Heat-induced antigen retrieval was carried out by boiling sections in 0.01 M citrate buffer (pH 6.0). Washing steps were performed with PBS and membrane permeabilization was done with 0.01 M phosphate-buffered saline (PBS) including 0.1% Tergitol. Blocking of non-specific binding sites was achieved by incubating sections for 1 h in PBS incl. 4% milk powder and 10% fetal calf serum (FCS). The following primary antibodies were diluted in PBS supplemented with 10% FCS and incubated overnight at 4 °C in a humid chamber: IBA1 (recombinant guinea-pig IgG, 1 µg/ml, #234308, Synaptic Systems, Göttingen, Germany) and CD68 (monoclonal rabbit antibody, 1:500, E3O7V, Cell Signaling, Danvers, MA, USA). The following fluorescently labeled, secondary antibodies were likewise prepared and incubated for 1.5 h at 37 °C in the dark: goat anti-guinea pig (H + L) Alexa Fluor-488 (0.67 µg/ml, polyclonal, #A11073, ThermoFisher Scientific) and goat anti-rabbit (H + L) Alexa Fluor-555 (0.67 µg/ml, polyclonal, #A21428, ThermoFisher Scientific Invitrogen). Sections were mounted with 1 drop of Fluoroshield mounting medium (Merck, Darmstadt, Germany) containing DAPI, covered with coverslips and sealed with nail polish. Fluorescent images were taken with a TiE microscope (Nikon, Beverwijk, The Netherlands) and processed with NIS Elements Imaging software (Nikon).

### Quantification of microglial cells and phagocytosis

Large-scale images with IBA1 and DAPI signal were loaded into ImageJ to create a channel overlay image. Areas of interest were prepared for the cortex and hippocampus to exclude other regions. Individual microglia cells, defined by their positivity for IBA1 and DAPI nuclei signal, were labeled and counted via the multi-point function. For statistical analysis, the means of two sections per animal were used per region.

Assessing microglial phagocytosis via the extent of CD68 positivity in mouse brain sections was carried out using a custom ImageJ macro with the experimenter being blinded to the genotype of the sample. In brief, the threshold function was applied for both the CD68 and IBA1 signal to create selection masks for the two channels. Regions of interest of the different brain regions such as the hippocampus and cortex were defined and within each brain region, the overlap area between the CD68 selection mask and the IBA1 selection mask was determined. By subtracting the overlap from the total microglia area multiplied by 100, the CD68-IBA1 overlap per each brain region in percentage was calculated.

### Bulk hippocampal transcriptome analysis

Total RNA sequencing libraries were prepared using the Illumina Stranded Total RNA Prep, Ligation with Ribo-Zero Plus Kit (#20040525) with 500 ng RNA as input. RNA quality and quantity were assessed on the Bioanalyzer (Agilent 2100, RNA 6000 Nano Kit #*5067 − 1511*) with Qubit 3.0 Fluorometer (RNA High Sensitivity Assay Kit #Q32852). Library amplification was performed with 12 PCR cycles, and multiplexing was achieved using the IDT for Illumina RNA UD Indexes Set B, Ligation (#20040554) Kit. Library quality was validated on the Bioanalyzer (Agilent 2100, High Sensitivity DNA Kit #*5067 − 4626*). Sequencing was performed on a NextSeq 2000 (Illumina) using a 50 bp single-read configuration.

Raw sequencing reads were processed and demultiplexed using bcl2fastq2 (v2.20.0). The quality of raw sequencing data was assessed with FastQC (v0.11.9). Reads were aligned to the mouse mm10 genome using the STAR aligner (v2.7.3a), and read counts were determined with featureCounts (v1.5.1). Data normalization and differential expression analysis were performed using DESeq2 (v1.44.0) [35] with correction for one unknown confounder by RUVSeq (v1.38.0) [36]. Low-count genes with a basemean < 10 were excluded from the analysis including volcano plot visualization. GO term analysis and pathway enrichment was performed using clusterProfiler (v4.12.3) [37].

### Quantitative proteome analysis of hippocampal protein extracts

Soluble hippocampal protein extracts were generated as previously described [38]. In brief, deep-frozen hippocampi from female animals were homogenized in 120 µl Tris-buffered saline (TBS, 120 mM NaCl, 50 mM Tris, pH 8.0 including phosphate (PhosSTOP) and phosphatase inhibitors (Complete mini) from Roche) per 10 mg tissue in a glass-teflon homogenizer. The homogenates were centrifuged at 17,000 × g for 20 min at 4 °C, and the supernatants containing TBS-soluble proteins stored at − 80 °C until further use.

Proteome analysis of TBS-soluble hippocampal protein extracts was performed as originally established for synaptic protein fractions [39] and recently adapted to purified myelin [40] and optic nerve lysate [41]. Briefly, TBS extracts corresponding to 20 µg protein were dissolved in lysis buffer (7 M urea, 2 M thiourea, 10 mM DTT, 2% CHAPS, 0.1 M Tris, pH 8.5). After removal of the detergents and protein alkylation, proteins were digested overnight at 37 °C with 400 ng trypsin. Tryptic peptides were directly subjected to LC-MS analysis. For quantification according to the TOP3 approach [42], aliquots were spiked with 10 fmol/µl of Hi3 EColi standard (Waters Corporation, Milford, MA, USA), containing a set of quantified synthetic peptides derived from *E. coli*. Peptide separation by nanoscale reversed-phase UPLC was performed on a nanoAcquity system (Waters Corporation), as described [41]. Mass spectrometric analysis on a quadrupole time-of-flight mass spectrometer with an ion mobility option (Synapt G2-S, Waters Corporation) was performed in an ion mobility-enhanced DIA mode with drift time-specific collision energies, referred to as UDMS^E^ [43]. Processing of LC-MS data and a searching against UniProtKB/Swiss-Prot mouse proteome database (release 2022_05, 17137 entries) were performed using the Waters ProteinLynx Global Server (PLGS) version 3.0.3 with published settings [41]. For post-identification analysis including TOP3 quantification of proteins, the freely available software ISOQuant [43] was used. False discovery rate for both peptides and proteins was set to 1% threshold and only proteins reported by at least two peptides (one of which having been unique) were quantified as parts per million (ppm) abundance values (i.e. the relative amount (w/w) of each protein in respect to the sum over all detected proteins). Proteins identified as contaminants from blood (albumin, hemoglobin) or skin/hair cells (keratins) were removed and potential outlier proteins were revised by inspection of the quality of peptide identification, quantification and distribution between protein isoforms. TBS-soluble hippocampal protein extracts from five animals per condition (WT, STAT1^−/−^) were processed with replicate injection, resulting in two technical replicates per biological replicate and, thus, in a total of 20 LC-MS runs to be compared. The Bioconductor R package ‘limma’ [44] was used to estimate the correlation between injection replicates with the function ‘duplicate correction’ [45] and to detect significant changes in protein abundance by moderated *t*-statistics [46]. The Bioconductor R package ‘q-value’ [47] was used to correct for multiple testing. For visualization of the entire proteome dataset, Pearson’s correlation coefficients derived from log_2_-transformed ppm abundance values were clustered and visualized with the tool heatmap.2 contained in the R package ‘gplots’. Only pairwise complete observations were considered to reduce the influence of missing values on clustering behavior.

### Statistical analysis

Depending on the experiments, unpaired *t* tests, multiple *t* tests, one-way analysis of variance (ANOVA) or two-way analysis of variance followed by specific post tests were applied. All data are displayed as means ± standard deviations (SD). Significant differences were marked as follows: *p < 0.05, **p < 0.01, ***p < 0.001, ****p < 0.0001. All calculations were conducted with Graphpad Prism Version 10.3.1 for Windows (Graph Pad Software, San Diego, USA).

## Results

### STAT1^−/−^ mice do not show altered motor performance or anxiety behavior

**Fig. 2:**
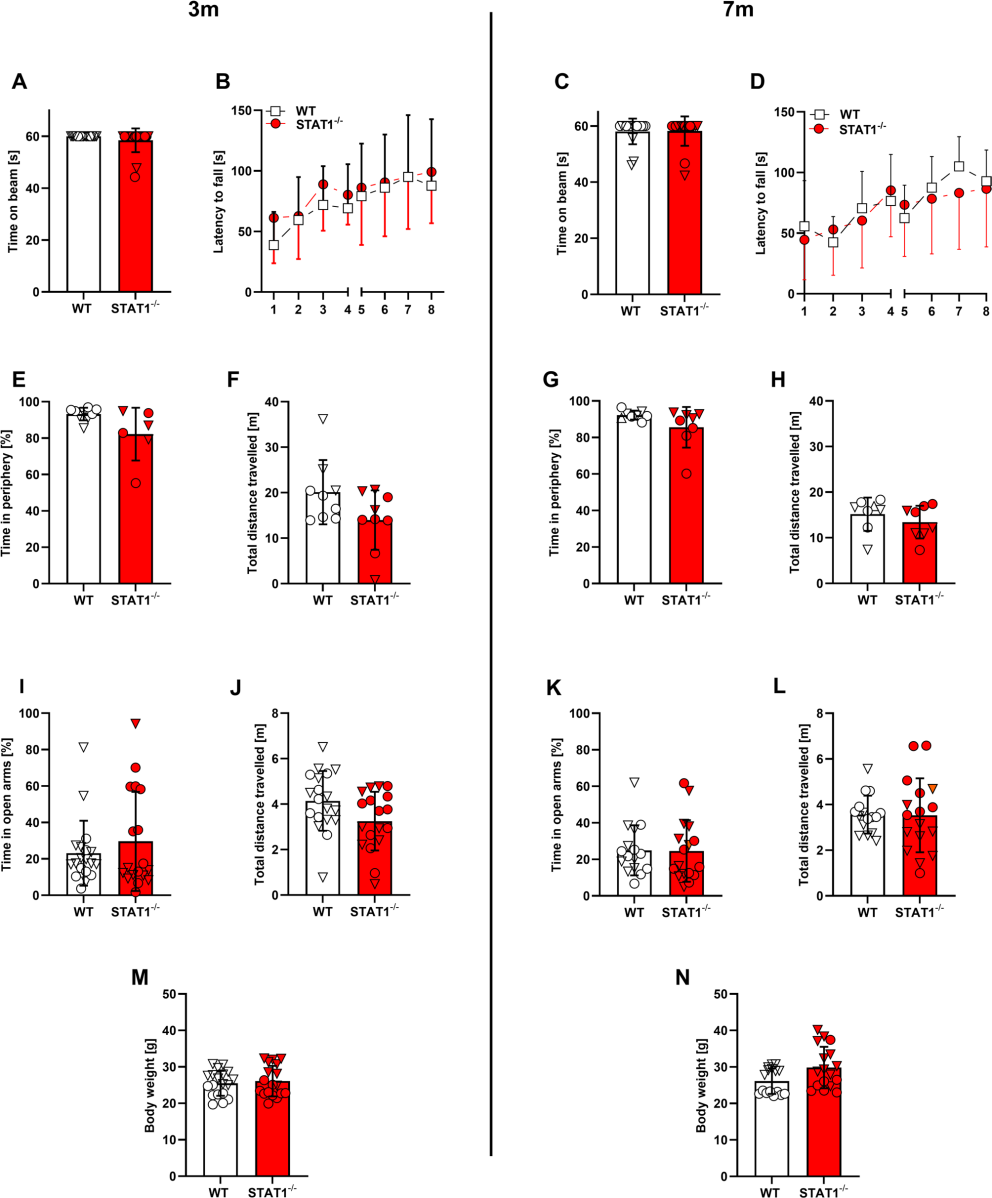
Motor and anxiety phenotype remained unaffected by the STAT1 deletion at 3 and 7 months of age. Female (▽) and male (○) WT and STAT1^-/-^ mice were subjected to the balance beam (**A, C**), accelerating rotarod (**B, D**), open field (**E-H**) and elevated plus maze tests (**I-L**) at an age of 3 and 7 months (*n* = 16–19). Body weights were unaffected by the expression of truncated STAT1 (**M, N**). No significant differences were observed in any of the motor or anxiety tests at either age. All data are given as means ± SD. Unpaired t test

The balance beam and accelerating rotarod tests were used to assess motor performances in 3- and 7-month-old animals (Fig. 2A-D). At each age, WT and STAT1^−/−^ mice performed in a comparable manner in each of the motor tasks. In the balance beam test, almost all animals of both genotypes managed to stay on the beam for the entire 60 s or were able to reach one of the escape platforms (Fig. 2A, C). In the rotarod test, both genotypes started with a latency to fall at around 50 s and increased their latency to on average 100 s on the second day (Fig. 2B, D). In order to investigate an altered anxiety behavior, the open field (Fig. 2E-H) and elevated plus maze tests were carried out (Fig. 2I-L). No differences among the groups were revealed in either of the tests. In addition, no differences in body weight between WT and STAT1^−/−^ mice were detected at either time point (Fig. 2M, N).

### No alterations in spatial or object recognition memory in STAT1^−/−^ mice

**Fig. 3:**
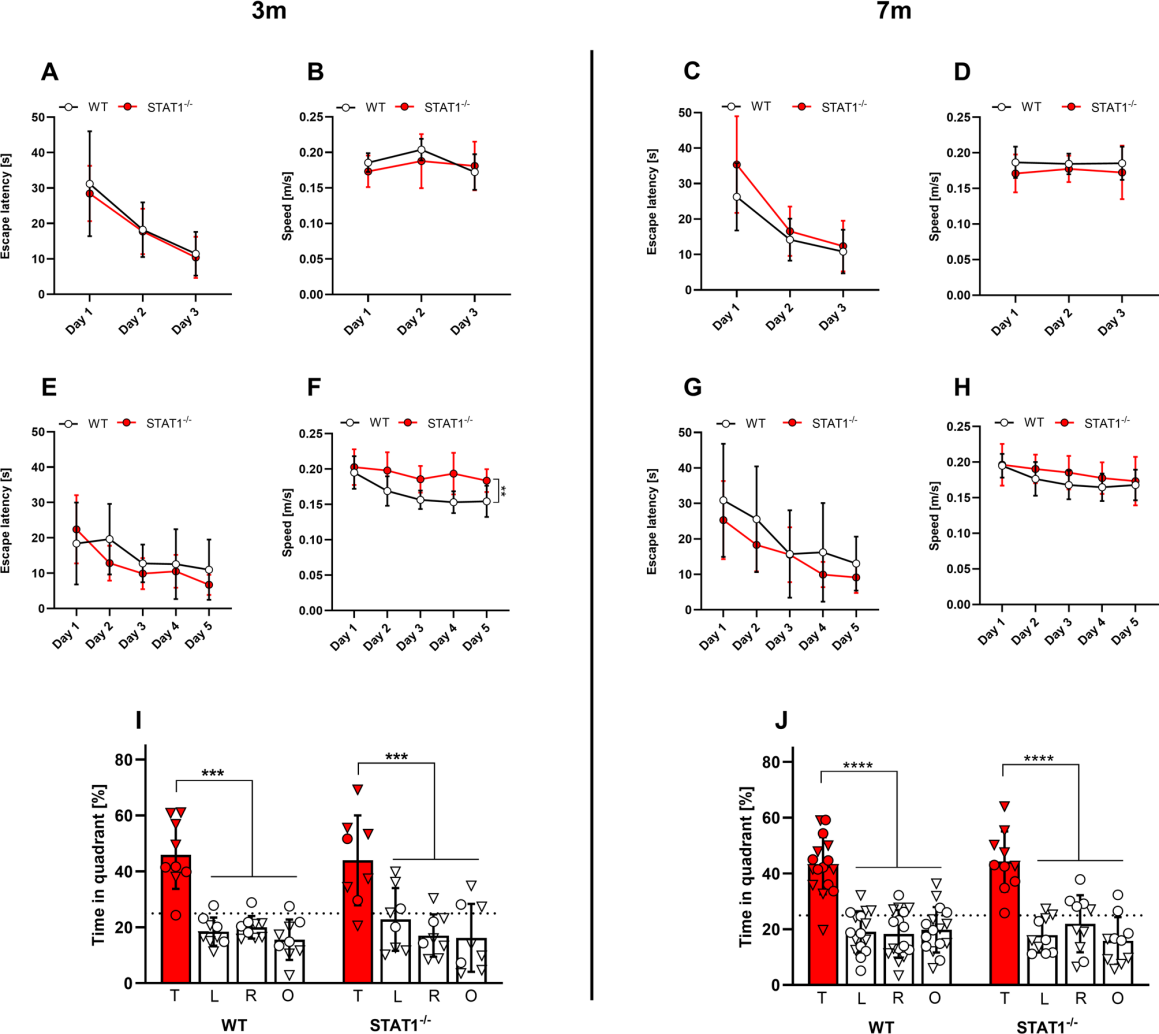
Spatial memory in the Morris water maze remained unaffected by the expression of a dysfunctional STAT1 mutant at 3 and 7 months of age. No significant differences were detected for the escape latency during cued (**A-D**) and acquisition tests (**E-H**) at any age. Three-month-old STAT1^-/-^ mice swam significantly faster in the acquisition test (**F**) than their WT counterparts. At both ages, female (▽) and male (○) WT and STAT1^-/-^ mice spent significantly more time in the goal quadrant (**I, J**) during the probe trial, revealing an intact spatial memory. Two-way ANOVA followed by Bonferroni’s multiple comparison test. T – target quadrant, L – left quadrant, R – right quadrant, O – opposite quadrant. All data are expressed as means ± SD. ** *p* < 0.01, *** *p* < 0.001, **** *p* < 0.0001

Spatial memory was tested in the cross maze and MWM tasks, while object recognition memory was evaluated using the novel object recognition test. Although 7-month-old STAT1^−/−^ mice started with a slightly higher escape latency than the WT group in the cued training of the Morris water maze (Fig. 3C, D), both groups managed to decrease their latencies to approximately 10 s on day 3. At 3 months of age, both groups started at ~ 30 s latency and progressively decreased it to ~ 10 s (Fig. 3A, B). During the subsequent acquisition training, both groups at the age of 7 months started with an escape latency of ~ 30 s and improved progressively to ~ 10 s on day 5, revealing no differences between the genotypes (Fig. 3G, H). Three-month-old mice, however, started at ~ 20 s escape latency and likewise improved to ~ 10 s on day 5 (Fig. 3E, F). During the probe trial, both genotypes spent an equal amount of time in the goal quadrant, revealing no differences among the groups (Fig. 3I, J). While no differences in swimming speed were detected in 7-month-old mice, young STAT1^−/−^ mice were significantly faster (0.2 m/s) than WT control mice (0.15 m/s) during the acquisition training (Fig. 3F, H).

**Fig. 4:**
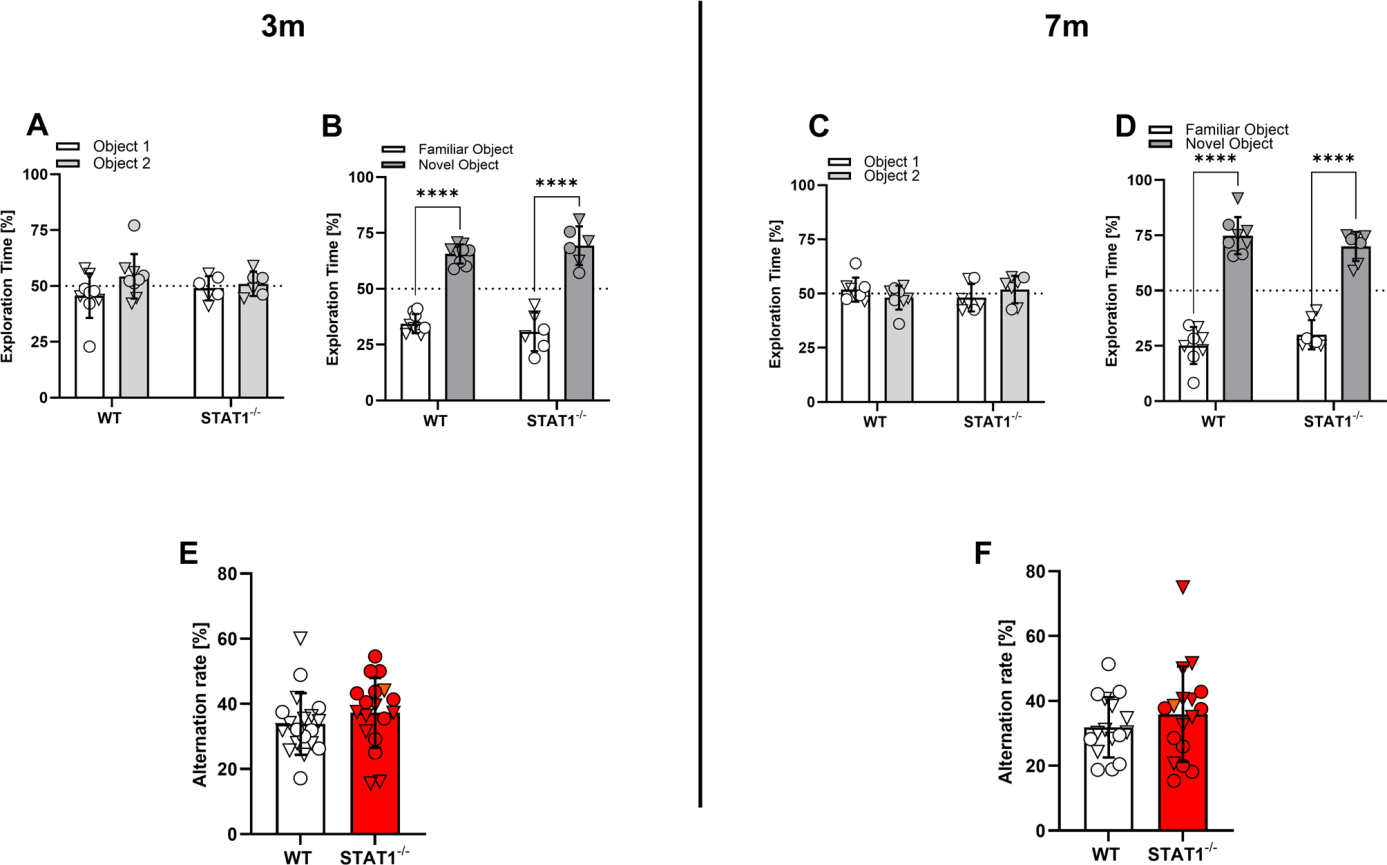
Novel object recognition and spatial memory (cross-maze) were unaffected by the STAT1 deletion at 3 and 7 months of age. Female (▽) and male (○) mice of both genotypes presented a significant preference for the novel object at both time points in the novel object recognition task (**A-D**). No differences in alternation rates were found among the groups in the cross-maze task at either age (**E-F**). Two-way ANOVA followed by Bonferroni’s multiple comparison test (**A-D**) and unpaired *t* test (**E-F**). All data are expressed as means ± SD. **** *p* < 0.0001

Recognition and working memory were further assessed using the novel object recognition (Fig. 4A-D) and cross maze tasks (Fig. 4E, F), respectively. In the novel object recognition test, WT and STAT1^−/−^ mice both presented equally with a preference for the novel object at both ages tested, revealing no difference among the groups. Likewise, both groups presented with a similar alternation rate in the cross maze, again revealing no significant differences among groups in working memory assessment.

### Hippocampal gene expression changes identified by bulk transcriptome analysis

**Fig. 5:**
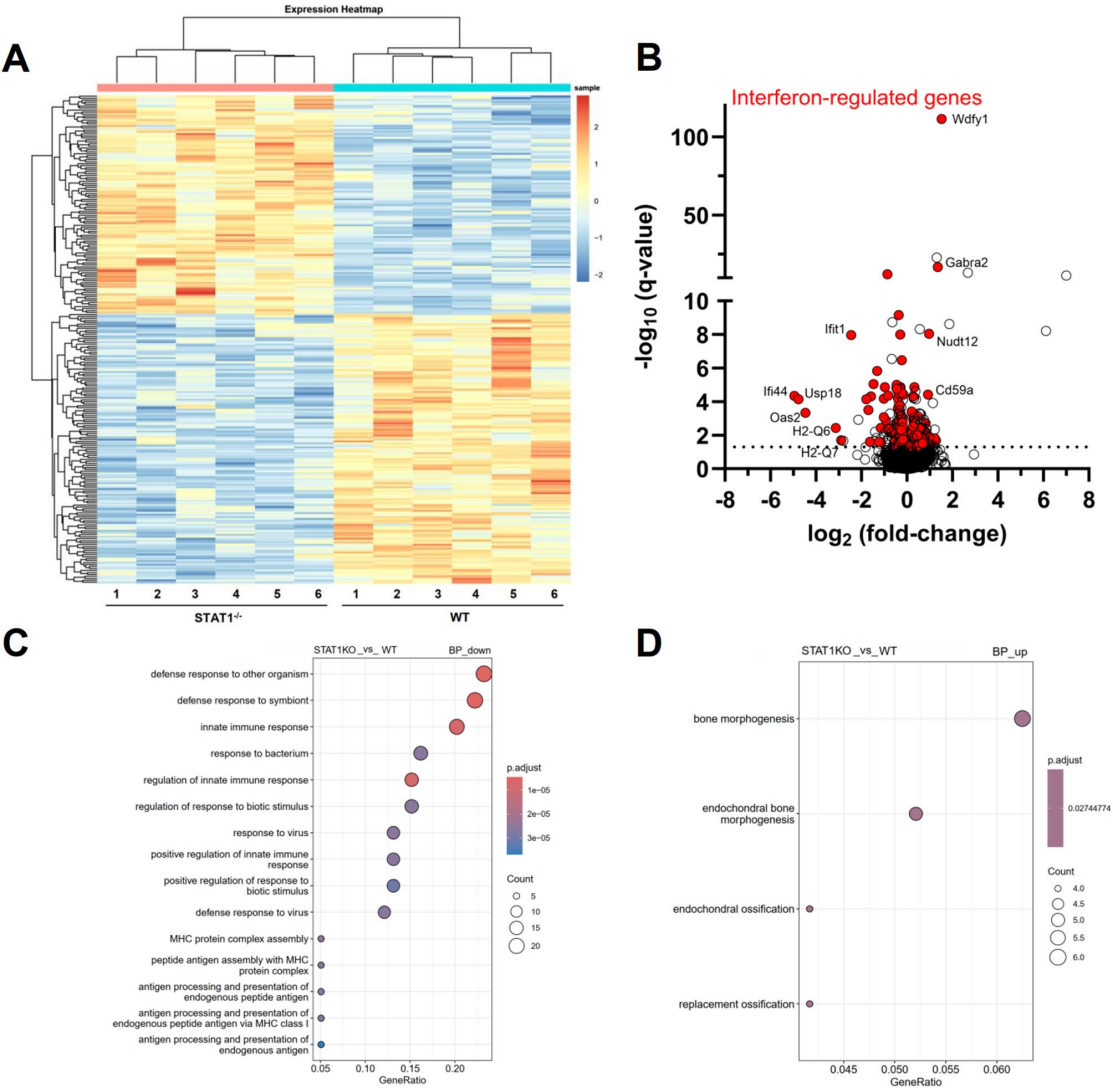
Bulk transcriptome analysis of hippocampal RNA from 7-month-old male WT and STAT1^-/-^ mice. Heatmap displaying differentially expressed genes (**A**). Down-regulated genes included mostly IFN-signaling-associated proteins such as *Irf9* and *Ifit1*. Up-regulated genes included several regulatory factors such as *CD59a* and *Serpina3n*. Volcano plot displaying the amount of differentially and not significantly differentially regulated genes (**B**). Gene Ontology enrichment analysis of differentially expressed genes in WT and STAT1^-/-^ mice identified significantly altered biological processes (BP) (**C**, down-regulated in STAT1^-/-^; **D**, up-regulated in STAT1^-/-^). The analysis was conducted using clusterProfiler (v4.12.3). Dot size corresponds to the number of genes per category, and color represents the adjusted p-value. Key enriched terms for the down-regulated genes in STAT1^-/-^ mice include defense response to pathogens and MHC protein complex processes. RNA from 6 animals per genotype was used. DEGs with an adjusted p-value (< 0.05) calculated with the Benjamini-Hochberg method were used as input. Color bar in A indicates z-score

We next carried out bulk RNA sequencing of the hippocampus in 7-month-old animals to investigate potential changes in gene expression due to STAT1 functional deficiency. Compared to WT, 179 transcripts were significantly up- and 202 were significantly down-regulated in STAT1^-/-^ mice (Fig. 5, Additional file 2). Looking at the identified biotypes in our study reveals that the majority of identified genes represents protein-coding genes (~ 80%). In addition, we identified a number of long non-coding RNAs, pseudogenes or experimentally to be confirmed transcripts (TECs) (indicated in Additional file 2).

Interestingly, by focusing only on the protein-coding genes, 135 of the down-regulated genes found in STAT1^−/−^ mouse brain bulk hippocampal transcriptome were identified as IFN-regulated genes (IRGs) according to the Interferome v2.01 database [48] and contained genes such as *IFN-regulatory factor 9* (*Irf9*), *IFN-induced protein with tetratricopeptide repeats 1* (*Ifit1*), and *IFN-regulatory factor 7* (*Irf7*) as well as genes associated with antiviral functions such as *galectin-3-binding protein* (*Lgals3bp*), *2’-5’-oligoadenylate synthetase* (*Oas2*), and *2’-5’-oligoadenylate synthetase-like 2* (*Oasl2*). In the group of the up-regulated genes, 72 protein-coding transcripts were identified as IRGs and included immune system regulatory factors such as *CD59a*, *Serpina3n* (reduces neuronal apoptosis and neuroinflammation [49, 50]), and *WD repeat and FYVE domain-containing protein 1* (*WDFY1*) as the most significantly regulated gene.

**Fig. 6:**
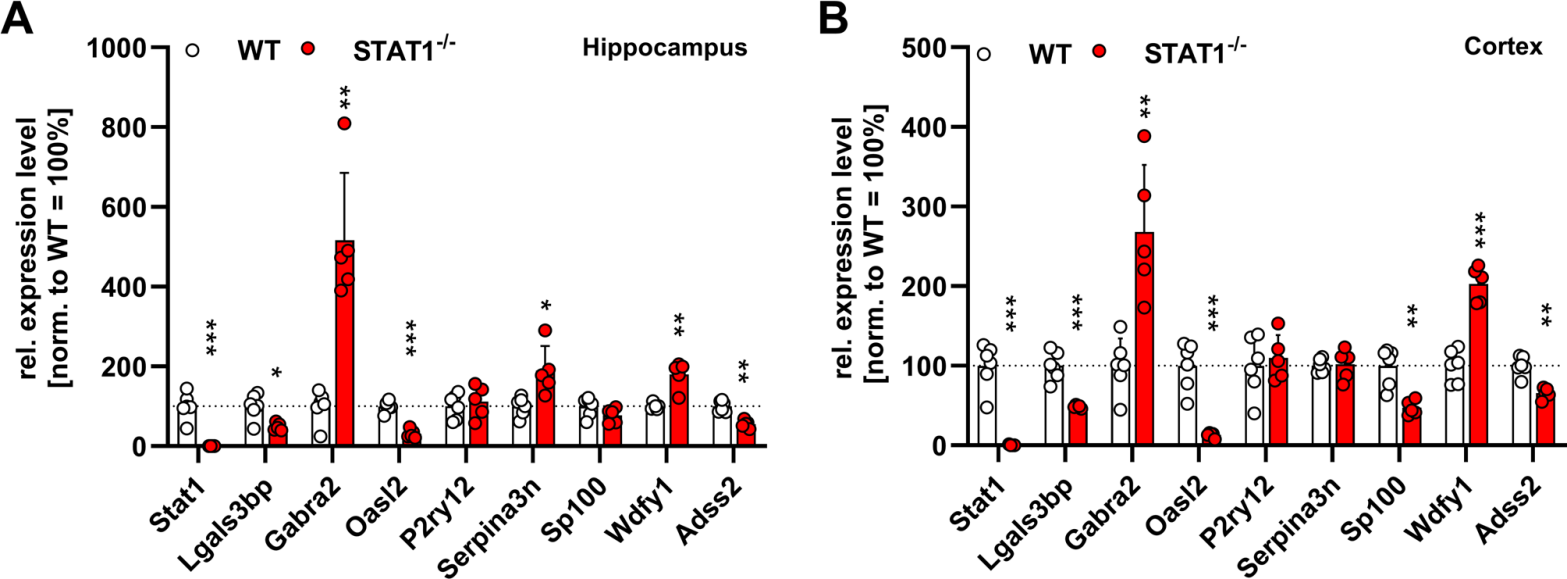
Validation of differentially expressed genes found in transcriptome analysis and selected microglial marker expression levels in the hippocampus (**A**) and cortex (**B**). All data are given as means ± SD (see Additional file 3 for results of multiple* t* tests). * *p* < 0.05, ** *p* < 0.01, *** *p* < 0.001

The functional absence of STAT1 was verified by a lack of the *STAT1* transcript via RT-qPCR analysis (Fig. 6A, B). To validate differentially expressed genes found in the bulk transcriptome analysis, RT-qPCR was conducted for selected genes associated with IFN signaling and immune activation. While the transcriptome analysis was restricted to the hippocampus, qPCR validation experiments were carried out in addition in cortical samples. Differential expression, as revealed by transcriptome analysis, was validated for *Lgals3bp*, *Oasl2*, *WDFY1*, *Serpina3n*,* Gabra2*, and *Adss2* (Fig. 5). While *Sp100* and *P2RY12* expression was found unchanged between WT and STAT1^−/−^ mice in the hippocampus, *Sp100* was significantly down-regulated in the cortex of STAT1^−/−^ mice (see Additional file 3 for statistical data).

### Proteome analysis of soluble hippocampal fractions from WT and STAT1^−/−^ mice

**Fig. 7:**
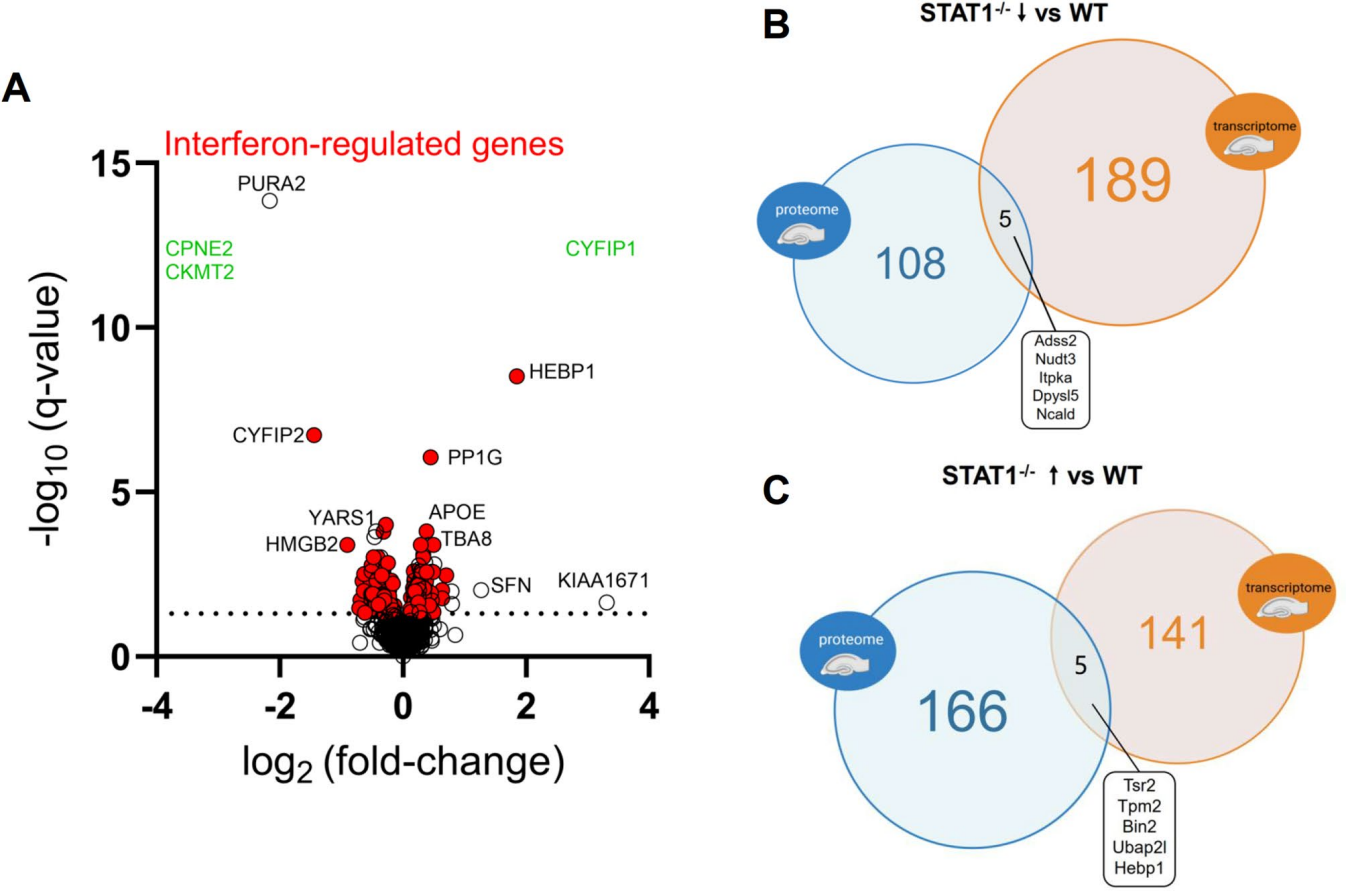
(**A**) Volcano plot illustrating the results from the proteome analysis (IFN-regulated genes according to Interferome 2.0 highlighted in red; Proteins found selectively in WT or STAT1^−/−^ are indicated in green). (**B, C**) Venn diagrams comparing significantly down- or up-regulated proteins with protein-coding genes from the transcriptome analysis

To gain insights into STAT1 deficiency-related expression changes also at the protein level, label-free protein quantification by liquid chromatography coupled to mass spectrometry (LC-MS) was applied to TBS-soluble hippocampal protein extracts from 7-month-old female WT and STAT1^−/−^ mice. Ion mobility-enhanced data-independent acquisition (DIA) mass spectrometry resulted in the quantification of 1406 proteins with a false discovery rate (FDR) of < 1% at peptide and protein level and an average sequence coverage of 37.5% (Additional file 4). A clustered heatmap of Pearson’s correlation coefficients for protein abundance showed that the LC-MS runs cluster with a high overall correlation (> 0.97) into two conditions defined by the genotype, in agreement with the experimental design (Additional file 5). Out of the quantified proteins, 166 were significantly up- and 108 significantly down-regulated in STAT1^−/−^ compared to WT control mice. A comparative analysis of the proteome data set (Fig. 7A) revealed that many of the significantly differentially regulated proteins were classified as IRGs (104 of the up- and 78 of the down-regulated proteins according to Interferome V2.01 [48]) and/or STAT1 target genes (8 of the top 10 regulated proteins according to the ENCODE transcription factor targets dataset [51]), underscoring the validity of our proteomic approach. A comparative analysis of down- and up-regulated protein sets and gene-coding transcripts from the transcriptome data set revealed an overlap of 5 and 5 genes/proteins respectively (Fig. 7B), including some of the strongest differentially regulated proteins such as HEBP1 or PURA2 (gene *Adss2*). Although we cannot exclude that the relatively small overlap of regulated genes/proteins may also reflect an influence of sex-related difference between transcriptome (male mice) and proteome (female mice), we favor the interpretation that this is rather an effect of the different analytical space: while our transcriptome analysis could cover all transcripts in principle, our proteome analysis was limited to a subfraction of proteins soluble in TBS in the absence of detergents.

### Unchanged microglia number and phagocytosis activation in older STAT1^−/−^mice

**Fig. 8:**
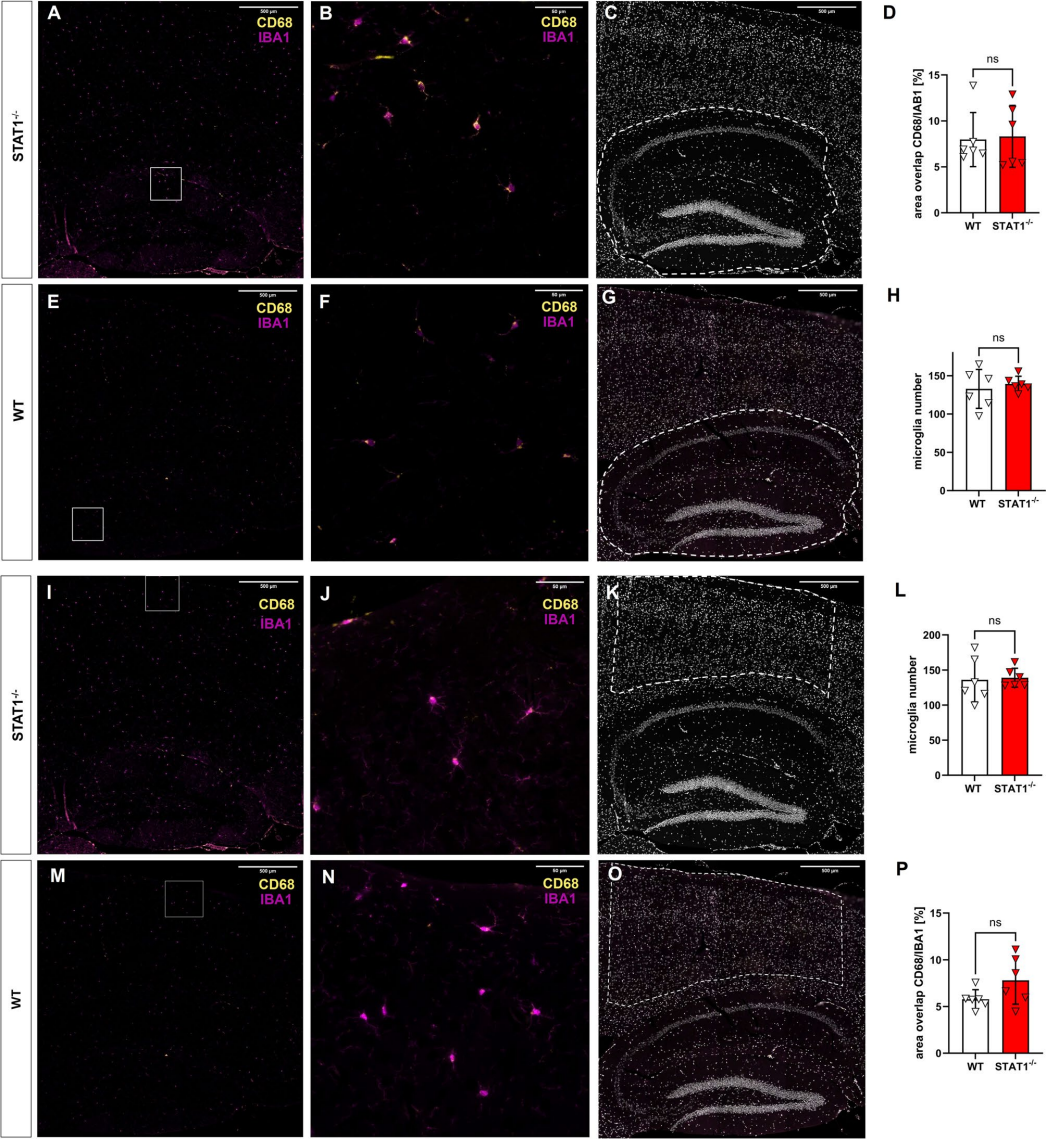
Fluorescent staining for IBA1 (magenta), CD68 (yellow) and DAPI (grey) in sagittal formalin-fixed, paraffin-embedded sections of the hippocampus (**A-C, E-G**) and cortex (**I-K, M-O**) from 7-month-old female STAT1^-/-^ and WT mice. Fluorescent image analysis (*n* = 6 animals, 2 sections per animal) revealed no difference in total microglia number or CD68/IBA1 area overlap in the hippocampal (**D, H**) or cortical (**L, P**) region. All data are given as means ± SD. Unpaired *t* test or multiple *t *tests. Scale bars: (**A, C, E, G, I, K, M, O**): 500 μm, (**B, F, J, N**): 50 μm

To investigate whether dysfunctional STAT1 signaling affects microglial proliferation and phagocytosis, fluorescent immunohistochemistry on formalin-fixed and paraffin-embedded brain sections using antibodies directed against the microglia marker IBA1 and the macrophage marker CD68 was performed. Total microglia numbers were quantified in the hippocampus and in the isocortex (regions were outlined as depicted in Fig. 8). Microglia were counted using the ImageJ multi-point and channels tool to overlay the microglia signal with the DAPI channel to distinguish individual cells. To evaluate phagocytosis, a self-built ImageJ macro was used to calculate the percentage of CD68/IBA1 signal overlap. No difference in microglia numbers was detected for WT (mean = 132.8) and STAT1^−/−^ (mean = 139.3) animals in the hippocampus (*p* = 0.57) (Fig. 7D). Likewise, microglia numbers remained unchanged between WT (mean = 135.9) and STAT1^−/−^ mice (mean = 139.9) in the isocortex (*p* = 0.83) (Fig. 7L). Calculation of the area of CD68/IBA1 overlap showed no significant differences for either the hippocampus (mean WT = 7.97%, mean STAT1^−/−^ = 8.32%; *p* = 0.852 (Fig. 7H)) or cortex (mean WT = 5.78%, mean STAT1^−/−^ = 7.8%; *p* = 0.101 (Fig. 7P)).

## Discussion

In order to investigate the impact of impaired STAT1 signaling on hippocampal functions, mice with a targeted disruption in the murine *Stat1* locus were investigated with respect to neurobehavioral alterations. Although no overt developmental abnormalities or alterations in growth, activity or reproductive abilities were described [29], a detailed behavioral analysis is lacking so far. In order to fill this gap, a comprehensive analysis of a variety of behavioral parameters was carried out in the present study. Previous studies investigating the effects of IFN-γ on cognition under normal conditions in the absence of a pathological stimulus, by either transgene-mediated subtle expression or IFN-γ knockout, reported improvements in spatial memory tasks in IFN-γ transgenic mice [13] and conversely memory deficits in IFN-γ KO mice under basal conditions [9], respectively. In contrast to such findings, we could not confirm impairments of the STAT1^−/−^ mice in any of the applied paradigms addressing anxiety, motor performance or learning and memory. Unlike in the studies mentioned, the STAT1^−/−^ mice used in the present study additively present with dysfunctional IFN-α signaling, which, if administered or overexpressed, has consistently been described as rather detrimental with respect to cognition both in humans and mice [15, 21, 52, 53]. Thus, a simultaneous suppression of IFN-α signaling might blunt any slight but measurable effects of inhibited IFN-γ signaling under baseline conditions in the analyzed mouse line. Quantification of IBA1-positive microglia and extent of CD68 positivity as marker for phagocytic activity did not result in any difference between the WT and STAT1^−/−^ mice. This may be explained by the absence of any stressor that would normally during onset of infection, trauma or pathology trigger activation and proliferation of resident microglia cells [23, 54, 55].

Analyses of gene expression changes revealed *WDFY1* to be one of the most significantly up-regulated genes in the STAT1^−/−^ background which was previously reported as up-regulated in STAT1-deficient murine mixed glial cell cultures [56]. WDFY1 plays a crucial role in the innate immune system as it acts as an adaptor molecule to recruit the signaling adaptor TRIF to the pattern recognition receptors TLR3 and TLR4 [57]. While an overexpression of *WDFY1* potentiates the activation of IFN-regulatory factor 3 (IRF3) and the production of inflammatory cytokines, a depletion of WDFY1 causes the opposite effect [58]. Although *Wdfy1* belongs to the group of IRGs, we cannot rule out that the observed difference in gene expression is at least partially affected by slight genetic differences in C57BL6 substrains. An analysis between C57BL6/J and C57BL6/N mice revealed that genes such as insulin-degrading enzyme (*Ide*), *Adss2*, ectonucleotide triphosphate diphosphohydrolase 4 (*Entpd4*), *Nnt*, *Wdfy1* and dynein light chain Tctex-type (*Dynlt1*) were differentially expressed in B6J and B6N mice in seven different tissues [59]. In addition, behavioral differences have been reported among C57BL6 substrains [60]. However, no apparent changes on the behavioral level were detected between WT and STAT1^−/−^ mice and only half of the genes mentioned before (*Ide*, *Adss2*, *Wdfy1*) were found to show altered expression levels in the current dataset.

*S100A6* (calcyclin) has a variety of functions with regard to cell stress and differentiation and is regulated by a variety of transcription factors [61]. It also plays a role in cell cycle entry and progression in endothelial cells and has been demonstrated to act as a suppressor of antiproliferative STAT1 signaling [62]. Among the most significantly down-regulated genes, *ubiquitin-specific protease 18* (*Usp18*), *IFN-induced protein 44* (*Ifi44*), *2’-5’-oligoadenylate synthetase 2* (*Oas2*), *2’-5’-oligoadenylate synthetase-like 2* (*Oasl2*) and *galectin-3-binding protein* (*Lgals3bp*) were found, which all code for proteins essentially involved in the innate immune response to viral infection. While microglial Usp18 has been demonstrated to negatively regulate the activation of STAT1 and thereby the induction of IFN-responsive genes [63], *Usp18* is also a STAT1 target gene according to the ENCODE transcription factor target data set [51, 64]. Both *Ifi44* and *Oas2* have been shown to be induced by unphosphorylated STAT1, which shows a slower decrease compared to phosphorylated STAT1 following IFN stimulation [65]. Validation by qPCR confirmed the strong down-regulation of *Oasl2* in both brain regions, which is in good agreement with in vitro data demonstrating a strong reduction in Oasl2 expression levels upon *STAT1* short interference RNA treatment of NIH-CUG2 cancer cells [66]. Ectopic expression of the human ortholog *OASL* in human cells has been shown to inhibit IFN induction through the cGAS-STING DNA-sensing pathway [67]. A recent study investigated transcriptional responses to IFN-γ in different brain cell types. Interestingly, *H2-Q6* and *H2-Q7*, which were both found to be significantly down-regulated in older STAT1^−/−^ mice, were robustly induced in microglia and to a lesser degree in neurons, but not in other glial cells such as oligodendrocytes or astrocytes [68].

We further identified heme-binding protein 1 (Hebp1) as one of the most strongly up-regulated proteins in the hippocampus in STAT1^−/−^ mice compared to WT controls, with *Hebp1* gene expression being also significantly up-regulated in our transcriptome data set, albeit to a lesser degree. Hebp1 is highly expressed in injury-associated microglia in a mouse model of spinal cord injury [69] as well as in neurons. It has been further described as a pre-symptomatic AD marker that is elevated in brains of the 3xTg transgenic AD mouse model and AD patients with a rapidly progressing form of the disease [70]. In good agreement with our observed up-regulation in mice with deficient STAT1 signaling, recent data indicate higher *Hebp1* gene expression in lungs of SARS-CoV2-infected hACE2;IFNAR1^−/−^ mice, which present with a blunted IFN-I response, compared to hACE2 mice [71]. Another down-regulated protein was the tyrosine tRNA synthetase SYYC (Yars1), which is also a STAT1 target gene. In gastric cancer cells, expression of the YARS protein is impaired by the STAT1 inhibitor fludarabine [72]. Interestingly, a deficiency in the complement regulator CD59a, a gene found to be strongly up-regulated in the STAT1^−/−^ mice, was shown to result in severe demyelination, axonal damage and inflammation after acute experimental allergic encephalomyelitis in mice [73]. Furthermore, CD59a is ubiquitously expressed on the cell surface, acting as a damage protector by inhibiting the formation of the membrane attack complex (MAC), a potent driver of inflammation, and, thereby, preventing activation of the complement cascade [74]. Given the complement system-activating properties of IFN-α, a direct or indirect negative regulation of CD59a by IFN-α and in consequence an activation of the MAC might be conceivable, albeit literature connecting IFN-α to CD59a directly is scarce. Hence, up-regulation of CD59a, blocking damages to CNS cells mediated by the complement system might be a protective mechanism under neuroinflammatory conditions. Further evidence for such a link is provided by another study on IFN-responsive oligodendrocytes in white matter aging, reporting that STAT1^+^ oligodendrocytes were always distinct from Serpina3n^+^ oligodendrocytes and devoid of any colocalization of the two proteins [75]. Although generally associated with neurotransmission and modulating various forms of behavior [76, 77], GABA_A_ receptors were additionally located on microglia and shown to possess a neuroprotective role and reduce cytokine-mediated damages when activated in inflammatory contexts [78]. Furthermore, it was shown that IFN-γ administration via intrathecal injections mimicking chronic IFN-γ exposure in vivo reduced GABAergic activity and neuronal inhibition [79]. Hence, IFN-γ might negatively regulate GABA-receptor expression in a direct or indirect fashion, explaining the observed strong up-regulation of *Gabra2* in the STAT1^−/−^ mice. However, the observed higher *Gabra2* levels in STAT1^−/−^ mice have to be interpreted with caution, as it must be noted that the C57BL6/J substrain carries a spontaneous intronic single nucleotide deletion causing decreased brain mRNA levels [80], which may have affected the observed expression differences.

In conclusion, the combined deficiency of IFN-α and IFN-γ signaling in STAT1^−/−^ mice under normal conditions neither leads to behavioral abnormalities, nor does it impact microglial proliferation or phagocytic activity. However, transcriptomic analysis revealed significant differential regulation of numerous inflammation-associated genes. As expected, genes involved in the innate immune response to viral infections were down-regulated, while in contrast, genes associated with anti-inflammatory and neuroprotective processes showed an up-regulation. These findings suggest a potential immune-modulating role of STAT1 deficiency, and future studies are needed to explore its impact in chronic, sterile neuroinflammatory conditions such as AD.

## Electronic supplementary material

Below is the link to the electronic supplementary material.

**Supplementary Material 1: Additional file 1:** Table summarizing the primer sets used in the present study

**Supplementary Material 2: Additional file 2:** Tables summarizing differentially up- and down-regulated genes in the hippocampus between STAT1^−/−^ and WT mice at 7 months of age

**Supplementary Material 3: Additional file 3:** Statistical analyses of RT-PCR validation of selected genes in the hippocampus and cortex between 7-month-old WT and STAT1^−/−^ mice

**Supplementary Material 4: Additional file 4:** Tables summarizing significantly up- and down-regulated proteins in the hippocampus between STAT1^-/-^ and WT mice at 7 months of age

**Supplementary Material 5: Additional file 5:** Clustered heatmap of Pearson’s correlation coefficients for protein abundance showing that the LC-MS runs cluster with a high overall correlation (> 0.97) into two conditions defined by the genotype, in agreement with the experimental design

## Data Availability

The datasets generated during and/or analyzed during the current study are included within this article and its additional files or available from the corresponding author on reasonable request. The mass spectrometry proteomics data have been deposited to the ProteomeXchange Consortium via the PRIDE partner repository [81] with the dataset identifier PXD062965.
