# Supplementary material for: Behavioral assessment and gene expression changes in a mouse model with dysfunctional STAT1 signaling
Source: Cell Commun Signal. 2025 Jul 1;23:305. doi: 10.1186/s12964-025-02313-w (PMC12210716; doi:10.1186/s12964-025-02313-w)
Supplement: Supplementary file 1 — Supplementary Material 1: Additional file 1: Table summarizing the primer sets used in the present study [file 12964_2025_2313_MOESM1_ESM.pdf]

Primer	Sequence	Amplicon size	Acc. No.
β-Actin-forward	ATGGAGGGGAATACAGCCC	147	NM_007393
β-Actin reverse	TTCTTTGCAGCTCCTTCGTT		
STAT1-forward	TGCACATGACTTGATCCTTCA	127	NM_009283
STAT1-reverse	AAGATTTTGGAAAATGCCCA		
Lgals3bp-forward	TGCTGGTTCCAGGGACTCAA	104	NM_011150
Lags3bp_reverse	CCACCGGCCTCTGTAGAAGA		
Serpina3n-forward	CAGATCCCAGCCATCAAGAG	90	NM_009252
Serpina3n-reverse	CTGGCAGCTGGCTGGTTT		
WDFY1-forward	TGTTTGCAGTATGATCTGGACAC	112	NM_027057
WDFY1-reverse	CCTTGAGGGTCGTGATAACTGA		
Sp100-forward	AGCTACAACCACAGTCCCCT	133	NM_013673
Sp100-reverse	TCCTGTCCTTTTCCGTCTTCTAA		
P2ry12-forward	TTCCTGGGGTTGATAACCATTG	150	NM_027571
P2ry12-reverse	GGTGAGAATCATGTTAGGCAGTG		
Oasl2-forward	TTGTGCGGAGGATCAGGTACT	95	NM_011854
Oasl2-reverse	TGATGGTGTCGCAGTCTTTGA		
Gabra2-forward	CTTTCCATTTTTGCCGAAAG	137	NM_008066
Gabra2-reverse	TGATAATCGGCTTAGACCAGG		
Adss2-forward	ACACGGGGTAGAGAATTTGGA	117	NM_007422
Adss2-reverse	GGTAAGGGCCAACGCAGTA		

Additional file 1: Primer sequence and amplicon size information
