# Supplementary material for: Behavioral assessment and gene expression changes in a mouse model with dysfunctional STAT1 signaling
Source: Cell Commun Signal. 2025 Jul 1;23:305. doi: 10.1186/s12964-025-02313-w (PMC12210716; doi:10.1186/s12964-025-02313-w)
Supplement: Supplementary file 3 — Supplementary Material 3: Additional file 3: Statistical analyses of RT-PCR validation of selected genes in the hippocampus and cortex between 7-month-old WT and STAT1−/− mice [file 12964_2025_2313_MOESM3_ESM.pdf]

Gene	*p* value	Mean of WT	Mean of $STAT1^{-/-}$	Difference	SE of difference	Adjusted *p* value
**Stat1**	0.000083	100.0	0.03034	99.97	14.81	0.000584
**Lgals3bp**	0.007385	100.0	48.89	51.11	14.86	0.029213
**Gabra2**	0.000228	100.0	516.4	-416.4	70.53	0.001367
**Oasl2**	0.000007	100.0	31.03	68.97	7.478	0.000056
**P2ry12**	0.586591	100.0	112.2	-12.22	21.68	0.586591
**Serpina3n**	0.009013	100.0	189.4	-89.43	26.98	0.029213
**Sp100**	0.118521	100.0	77.39	22.61	13.10	0.222994
**Wdfy1**	0.000335	100.0	180.3	-80.30	14.34	0.001674
**Adss2**	0.000195	100.0	55.56	44.44	7.370	0.001367

RT-PCR validation hippocampus; Multiple unpaired t tests

Gene	*p* value	Mean of WT	Mean of $STAT1^{-/-}$	Difference	SE of difference	Adjusted *p* value
**Stat1**	0.000029	100.0	0.3176	99.68	12.90	0.000204
**Lgals3bp**	0.000133	100.0	48.58	51.42	8.099	0.000719
**Gabra2**	0.001498	100.0	268.1	-168.1	37.40	0.004901
**Oasl2**	0.000120	100.0	13.10	86.90	13.50	0.000719
**P2ry12**	0.641958	100.0	109.8	-9.794	20.36	0.871806
**Serpina3n**	0.832877	100.0	101.9	-1.863	8.576	0.871806
**Sp100**	0.001228	100.0	47.22	52.78	11.39	0.004901
**Wdfy1**	0.000021	100.0	202.8	-102.8	12.73	0.000165
**Adss2**	0.000296	100.0	66.43	34.57	6.071	0.001481

RT-PCR validation cortex; Multiple unpaired t tests

Additional file 3: Statistical analyses of RT-PCR validation of selected genes in the hippocampus and cortex between 7-month-old WT and $STAT1^{-/-}$ mice
