# Supplementary material for: Behavioral assessment and gene expression changes in a mouse model with dysfunctional STAT1 signaling
Source: Cell Commun Signal. 2025 Jul 1;23:305. doi: 10.1186/s12964-025-02313-w (PMC12210716; doi:10.1186/s12964-025-02313-w)
Supplement: Supplementary file 5 — Supplementary Material 5: Additional file 5: Clustered heatmap of Pearson’s correlation coefficients for protein abundance showing that the LC-MS runs cluster with a high overall correlation (> 0.97) into two conditions defined by the genotype, in agreement with the experimental design [file 12964_2025_2313_MOESM5_ESM.pdf]

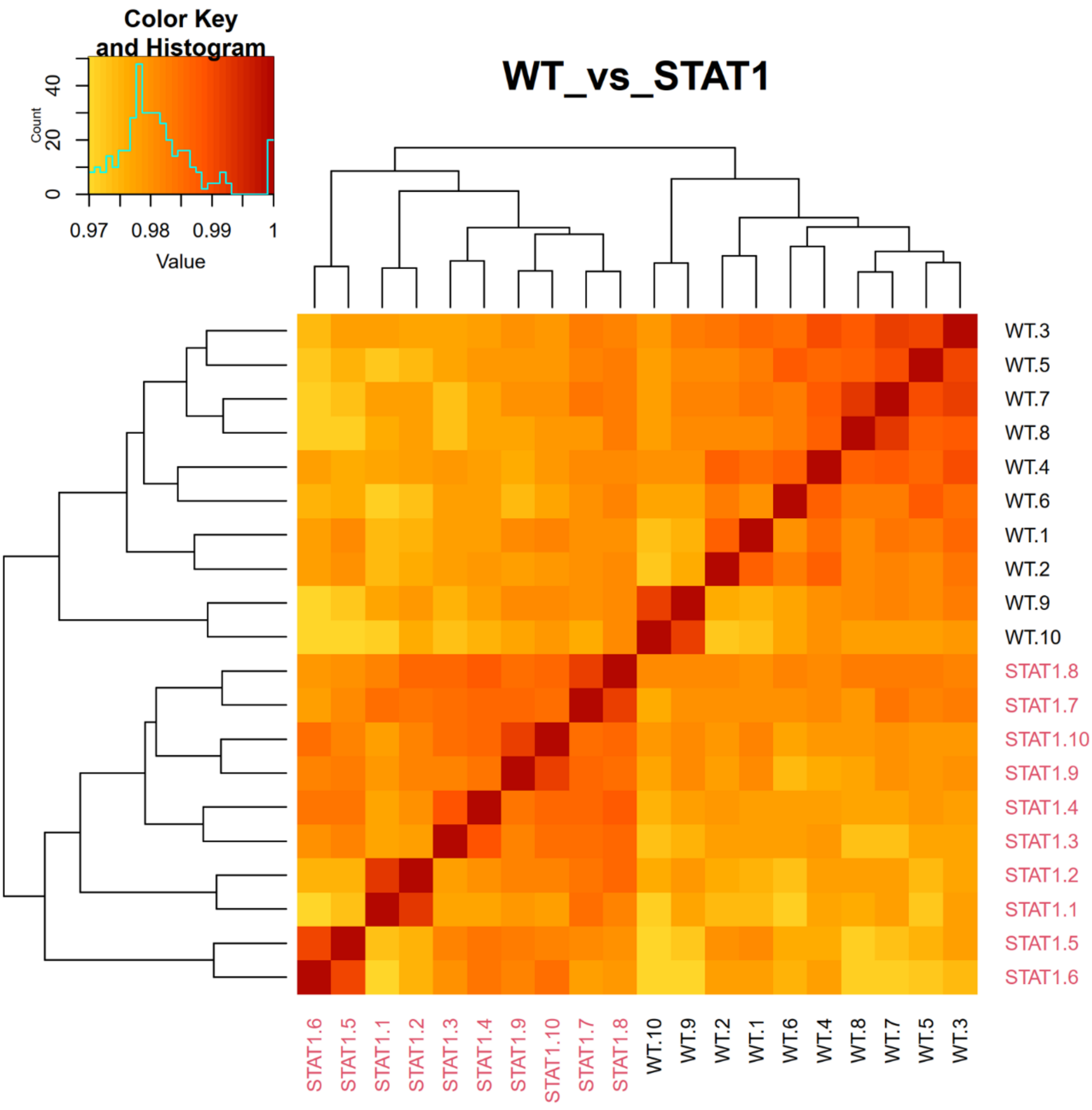

Additional file 5: Clustered heatmap of Pearson's correlation coefficients for protein abundance showing that the LC-MS runs cluster with a high overall correlation (>0.97) into two conditions defined by the genotype, in agreement with the experimental design
